# Lipoic Acid Attenuates Inflammation via cAMP and Protein Kinase A Signaling

**DOI:** 10.1371/journal.pone.0013058

**Published:** 2010-09-28

**Authors:** Sonemany Salinthone, Vijayshree Yadav, Robynn V. Schillace, Dennis N. Bourdette, Daniel W. Carr

**Affiliations:** 1 Portland Veterans Affairs Medical Center, Portland, Oregon, United States of America; 2 Department of Neurology, Oregon Health & Science University, Portland, Oregon, United States of America; 3 Department of Endocrinology, Oregon Health & Science University, Portland, Oregon, United States of America; University of Illinois at Chicago, United States of America

## Abstract

**Background:**

Abnormal regulation of the inflammatory response is an important component of diseases such as diabetes, Alzheimer's disease and multiple sclerosis (MS). Lipoic acid (LA) has been shown to have antioxidant and anti-inflammatory properties and is being pursued as a therapy for these diseases. We first reported that LA stimulates cAMP production via activation of G-protein coupled receptors and adenylyl cyclases. LA also suppressed NK cell activation and cytotoxicity. In this study we present evidence supporting the hypothesis that the anti-inflammatory properties of LA are mediated by the cAMP/PKA signaling cascade. Additionally, we show that LA oral administration elevates cAMP levels in MS subjects.

**Methodology/Principal Findings:**

We determined the effects of LA on IL-6, IL-17 and IL-10 secretion using ELISAs. Treatment with 50 µg/ml and 100 µg/ml LA significantly reduced IL-6 levels by 19 and 34%, respectively, in T cell enriched PBMCs. IL-17 levels were also reduced by 35 and 50%, respectively. Though not significant, LA appeared to have a biphasic effect on IL-10 production. Thymidine incorporation studies showed LA inhibited T cell proliferation by 90%. T-cell activation was reduced by 50% as measured by IL-2 secretion. Western blot analysis showed that LA treatment increased phosphorylation of Lck, a downstream effector of protein kinase A. Pretreatment with a peptide inhibitor of PKA, PKI, blocked LA inhibition of IL-2 and IFN gamma production, indicating that PKA mediates these responses. Oral administration of 1200 mg LA to MS subjects resulted in increased cAMP levels in PBMCs four hours after ingestion. Average cAMP levels in 20 subjects were 43% higher than baseline.

**Conclusions/Significance:**

Oral administration of LA *in vivo* resulted in significant increases in cAMP concentration. The anti-inflammatory effects of LA are mediated in part by the cAMP/PKA signaling cascade. These novel findings enhance our understanding of the mechanisms of action of LA.

## Introduction

Multiple sclerosis (MS) is a chronic disease of unknown etiology that affects the central nervous system (CNS) and is characterized by inflammation, demyelination and destruction of oligodendrocytes and axons. The immunology of MS is complex, involving autoreactive Th1 and Th17 lymphocytes, cells of the innate immune system including dendritic, natural killer (NK) and microglia cells, as well as vascular endothelial cells [1]. These cells produce an array of proinflammatory cytokines and chemokines such as interleukin (IL)-17, interferon (IFN)-γ, CCL5, CXCR3 and CXCL10 that contribute to disease pathogenesis [2]. Current FDA approved disease modifying therapies for MS such as beta interferon, glatiramer acetate and natalizumab are only partially effective and cause side-effects. Thus, there is a need for the continued development of new treatment strategies and targets for successful management of MS. There is increasing interest in the use of antioxidants, such as lipoic acid (LA), for the treatment of MS and other inflammatory diseases.

LA is a small molecule fatty acid that is endogenously synthesized and functions *in vivo* as a cofactor for many mitochondrial dehydrogenase enzymes such as pyruvate dehydrogenase [3], [4]. Exogenous sources of LA have been shown to exhibit both antioxidant and anti-inflammatory properties. LA inhibits vascular cell adhesion molecule (VCAM)-1 and intercellular adhesion molecule (ICAM)-1 production [5], [6], downregulates surface CD4 expression on blood mononuclear cells [7], attenuates tumor necrosis factor (TNF)-α and monocyte chemoattractant protein (MCP)-1 secretion [8], and inhibits NK cell activation and cytotoxicity [9]. Recently, Marracci *et al.* showed that LA suppresses experimental autoimmune encephalomyelitis (EAE) in SJL mice [10]. Two independent groups have since reported similar findings in both mouse and rat models of EAE [11], [12]. These investigations showed reduced T cell and macrophage infiltration into the CNS and spinal cords, decreased demyelination and axonal damage, reduction in both Th1 and Th2 cytokines including IL-4 and IFNγ, inhibition of matrix metalloproteinase (MMP) 2 and MMP9 proteolytic activity, reduced ICAM-1 and VCAM-1 expression, and prevention of blood brain barrier permeability [5], [10], [11], [12], [13]. Furthermore, a pilot study by Yadav *et al.* demonstrated that oral ingestion of LA is safe in MS subjects and resulted in reduced serum MMP9 and sICAM-1 levels [14]. However, the cellular and biochemical mechanisms that mediate the anti-inflammatory effects of LA are not fully understood.

We first reported the novel finding that LA stimulates cAMP production in human T lymphocytes, NK cells and PBMCs *in vitro* [9], [15]. It is well documented that cAMP is able to act as an immunomodulator. Activation of the cAMP dependent signaling pathway may contribute to the generation of Th2 lymphocytes by providing a strong inhibitory signal for Th1 cytokines, whereas Th2 cytokines are either unaffected or upregulated by this signal transduction system [16]. For example, increased cAMP levels stimulated by phorbol-myristate acetate plus the calcium ionophore A23187 in T cell lines had no effect on IL-4 mRNA expression, but inhibited IL-2 mRNA expression [17]. Similarly, increased cAMP levels in Th2 and Th0 clones treated with prostaglandin E_2_ (PGE_2_) had no effect on IL-4 production while IL-5 synthesis was inhibited. In contrast, production of IFNγ and IL-2 from Th1 clones were consistently inhibited by PGE_2_ [18], [19].

cAMP is a small molecule second messenger mediating signal transduction initiated by ligand binding to G-protein coupled receptors, such as histamine, prostaglandin and adrenergic receptors, and subsequent activation of adenylyl cyclases. cAMP activates protein kinase A (PKA) by binding to the regulatory subunits of PKA, releasing the catalytic subunits from inhibition, and allowing the phosphorylation of various downstream targets, including Lck [20]. Due to the large number of ligands and GPCRs available, cAMP, via activation of PKA, is involved in regulating many physiological and pathophysiological processes.

In this study, we present evidence demonstrating that LA treatment inhibited IL-6 and IL-17 production and decreased T cell proliferation and activation. LA appeared to have a biphasic effect on IL-10 secretion, however the data is not statistically significant. We also show that incubation with LA resulted in activation of PKA signaling and that blocking PKA using PKI inhibited LA mediated suppression of T cell and NK cell activation. Furthermore, we show for the first time that cAMP levels are elevated in PBMCs obtained from MS subjects four hours after oral ingestion of 1200 mg LA.

## Results

### LA reduces IL-6 and IL-17 secretion

**Figure 1:**
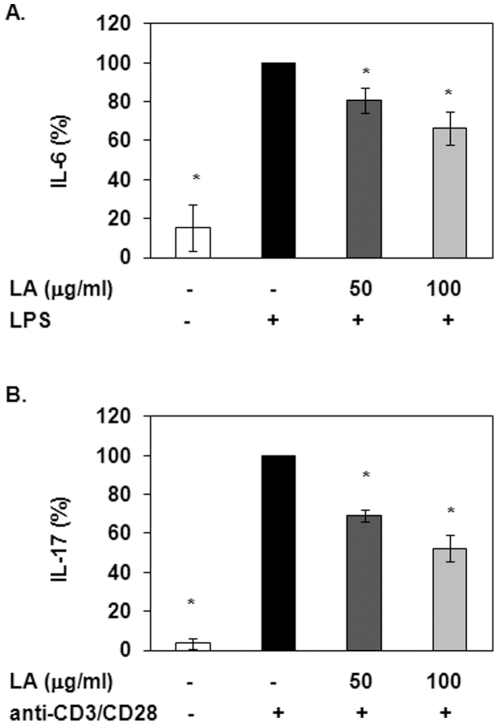
LA treatment suppresses the production of cytokines involved in Th17 T cell differentiation and activation. (A) T cell enriched PBMCs were pretreated with 50 or 100 µg/ml LA for 5 minutes and then stimulated with 5 µg/ml LPS for 6 hours. The supernatants were used to measure IL-6 levels via ELISA (R&D Systems, Minneapolis, MN). N = 3 donors in triplicate. * indicates significance compared to LPS control (*p*<0.05). (B) T cell enriched PBMCs were pretreated with LA prior to stimulation with a combination of 4 µg/ml anti-CD3 and 2 µg/ml anti-CD28 for 24 hours. Supernatants were used to measure IL-17 levels (eBiosciences, San Diego, CA). Depicted is a representative of 3 donors in triplicate. * indicates statistical significance compared to anti-CD3/CD28 control (*p*<0.05).

Naïve CD4^+^ T helper (Th) cells are able to undergo differentiation to specific lineages depending on the local cytokine environment. Recent studies in EAE suggest that Th17 cells play a crucial role in the pathogenesis of the disease [21]. We and others have shown that LA has anti-inflammatory properties in part by inhibiting production of some pro-inflammatory cytokines [5], [6], [8], [9]. However, it is not known how LA treatment will affect the synthesis of interleukin (IL)-6 and IL-17, which are involved in Th17 cell differentiation and function. We show that LA reduces IL-6 production in a dose-dependent manner (Fig. 1A). T cell enriched PBMCs were pretreated with 0, 50 or 100 µg/ml LA for 5 minutes prior to stimulation with 5 µg/ml LPS for 6 hours at 37°C. Supernatants were collected and used to measure IL-6 levels via ELISA. Treatment with LPS significantly increased IL-6 production compared to untreated controls. Pretreatment with 50 and 100 µg/ml LA prior to LPS stimulation resulted in reduced IL-6 levels by 19 and 34%, respectively.

IL-17 levels were also reduced upon pre-incubation with LA (Fig. 1B). T cell enriched PBMCs were incubated with 0, 50 or 100 µg/ml LA for 5 minutes. Cells were then treated with 4 µg/ml anti-CD3 and 2 µg/ml anti-CD28 for 24 hours at 37°C and supernatants were collected for analysis. Stimulation with anti-CD3/CD28 resulted in dramatic and statistically significant increases in IL-17 levels compared to untreated controls. Pretreatment with 50 and 100 µg/ml LA inhibited IL-17 production by 35 and 50%, respectively. Collectively, these data indicate that LA exerts anti-inflammatory functions in part by inhibiting IL-6 and IL-17 synthesis.

### LA inhibits T cell proliferation and activation

**Figure 2:**
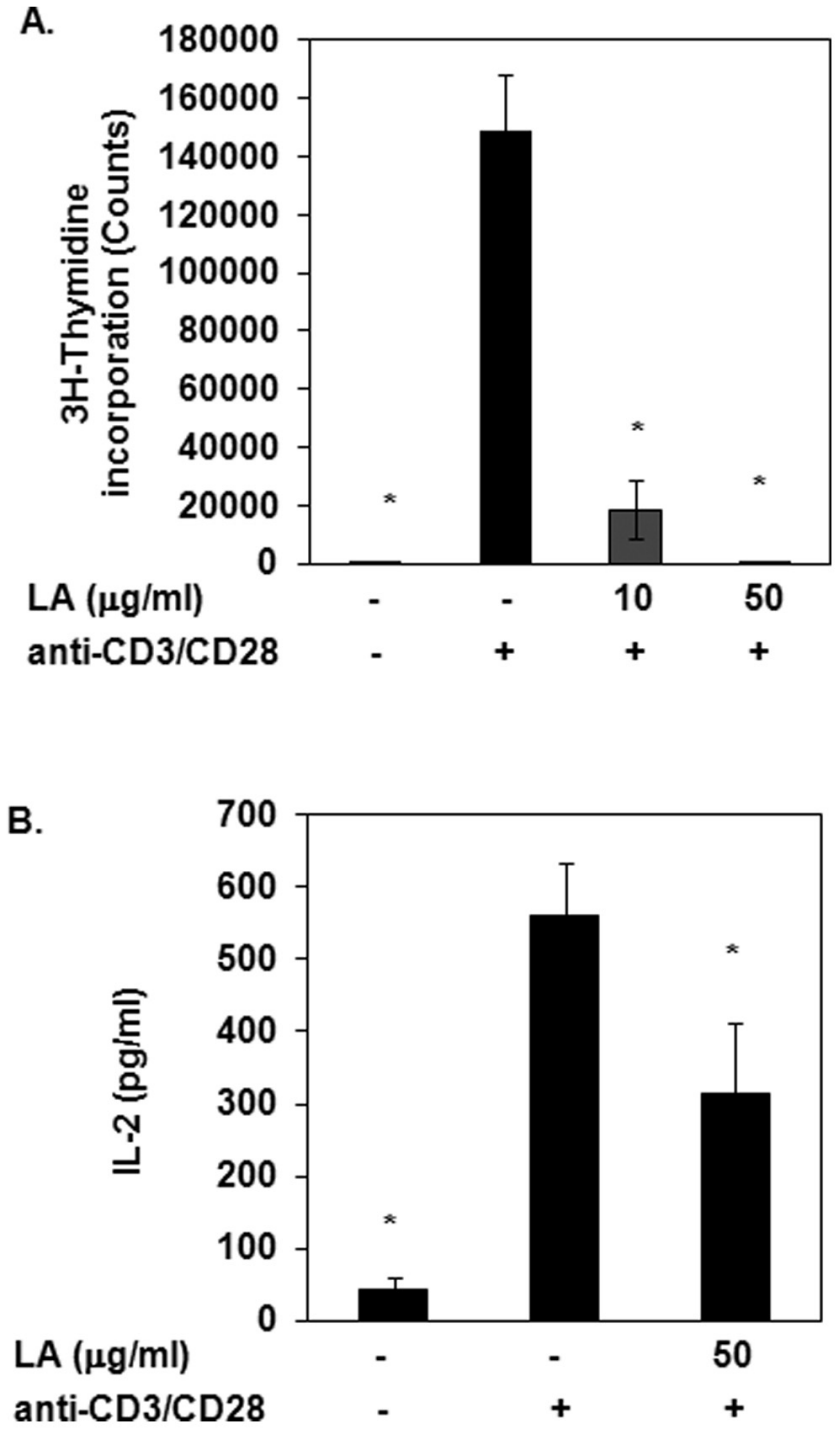
Treatment with LA attenuates T cell proliferation and activation. (A) T cell enriched PBMCs were pretreated with LA prior to stimulation with anti-CD3/CD28 for 72 hours. Proliferation was measured using 3H-Thymidine incorporation. N = 3. *indicates statistical significance compared to anti-CD3/CD28 control (*p*<0.05). (B) T cell enriched PBMCs were stimulated in triplicate with anti-CD3 (4 µg/ml, OKT3) and soluble anti-CD28 (5 µg/ml, BD Pharmingen) in the presence or absence of 50 µg/ml LA (1 minute pretreatment). Cells were then incubated for 6 hours at 37°C, 5% CO2. Supernatants were collected and IL-2 levels were analyzed by ELISA (R&D Systems, Minneapolis, MN). N = 3. *indicates statistical significance compared to anti-CD3/CD28 control (*p*<0.05).

LA prevents inflammatory T cell migration into the spinal cords of mice and rats with EAE [5], [12]. Here, we examined the effects of LA on human T cell proliferation and activation. T cell enriched PBMCs were incubated with LA prior to stimulation with anti-CD3 and anti-CD28. Proliferation was measured using ^3^H-thymidine incorporation while IL-2 production was used to measure T cell activation. Stimulation with anti-CD3/CD28 resulted in a significant and dramatic increase in ^3^H-thymidine incorporation compared to untreated cells (Fig. 2A). Pretreatment with 10 µg/ml LA inhibited ^3^H-thymidine incorporation by approximately 90% while 50 µg/ml LA completely abolished ^3^H-thymidine incorporation. Treatment with anti-CD3/CD28 also stimulated IL-2 production (Fig. 2B). Pretreatment with 50 µg/ml LA reduced IL-2 levels by approximately 50%. Collectively, the data presented here indicate that LA inhibits T cell proliferation and activation.

### Effect of LA on IL-10 production

**Figure 3:**
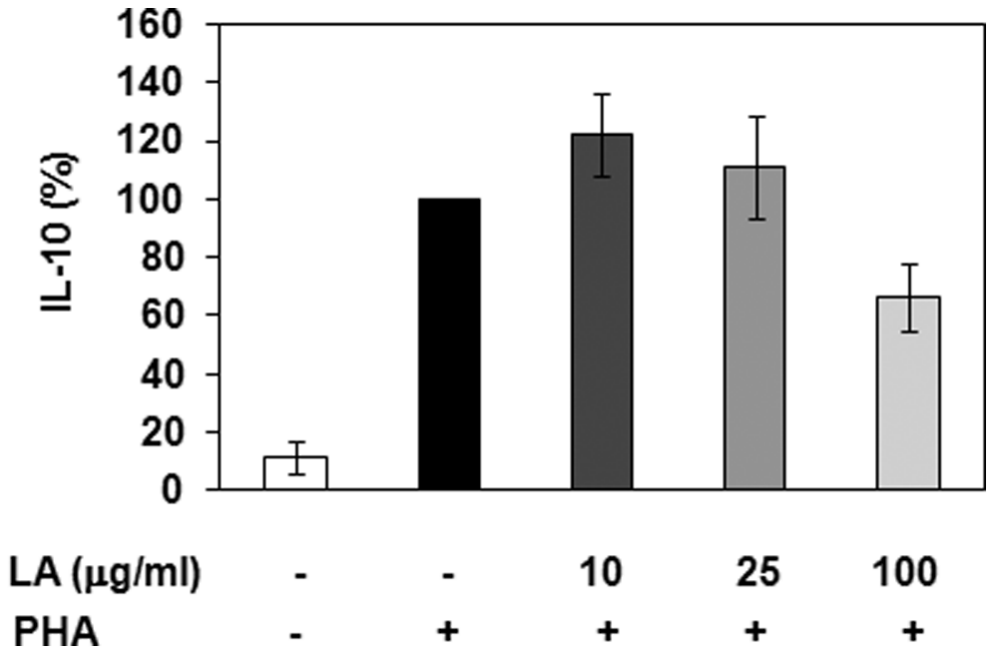
LA appears to have a biphasic effect on IL-10 production. T cell enriched PBMCs were treated with 10, 25, or 100 µg/ml LA for 5 minutes prior to treatment with 2 µg/ml PHA for 24 hours. Supernatants were used to measure IL-10 levels via ELISA (R&D systems, Minneapolis, MN). Percent change values were determined from PHA stimulated control. Depicted is the average of at least 3 donors in triplicate.

In the previous figures, we showed that LA inhibited production of pro-inflammatory cytokines. We next determined the effects of LA on production of the anti-inflammatory cytokine IL-10, which is produced by monocytes and lymphocytes and has been shown to be a mediator of the therapeutic effects of various treatments in EAE [22], [23]. IL-10 levels have also been shown to be elevated in the serum of MS patients after treatment with methylprednisolone [24]. There are reports that cAMP elevating agents increase IL-10 synthesis [25], [26], [27]. However, others have shown that cAMP decreases IL-10 production [28], [29]. To determine the effects of LA on IL-10 production, we first treated T cell enriched PBMCs with varying concentrations of LA, however, LA alone did not stimulate IL-10 production (data not shown). There are reports that the effects of cAMP elevating agents are only detected following T cell stimulation [30], [31]. As such, we determined the effects of LA on IL-10 synthesis after stimulation with PHA. T cell enriched PBMCs were incubated with 10, 25 or 100 µg/ml LA for 5 minutes then stimulated with 2 µg/ml PHA for 24 hours. The average basal IL-10 levels were 11.4% of PHA stimulated control (Fig. 3). The addition of LA resulted in a biphasic effect on IL-10 production, although the results are not statistically significant. In the presence of 10 or 25 µg/ml LA, PHA stimulation resulted in increased IL-10 levels compared to PHA only.. Treatment with 100 µg/ml LA appeared to decrease IL-10 levels. Collectively, the cytokine data suggest that LA is exerting its anti-inflammatory effects by lowering the concentrations of pro-inflammatory cytokines (IL-6 and IL-17) while possibly having a biphasic effect on IL-10.

### LA activates PKA signal transduction

**Figure 4:**
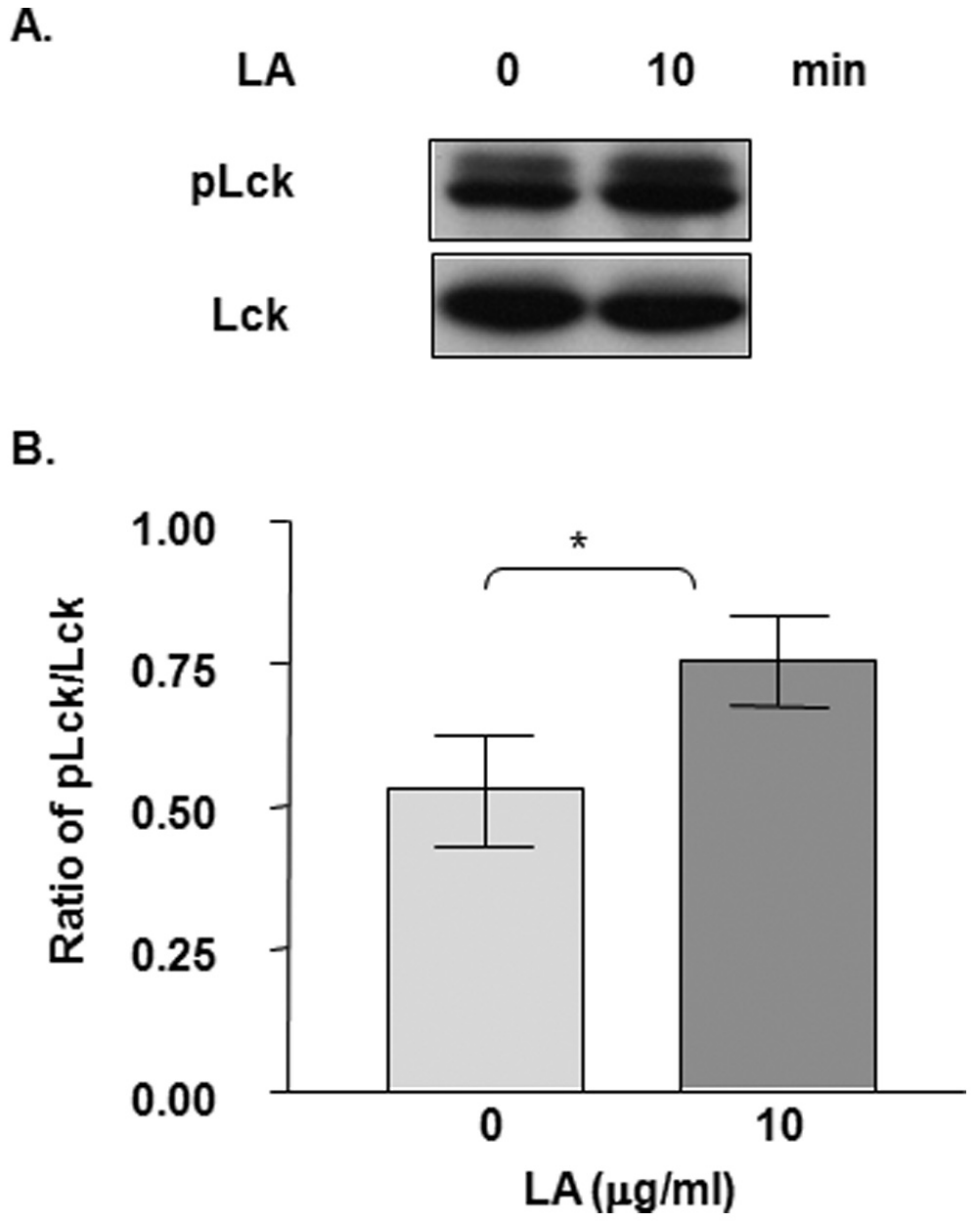
LA activates the protein kinase A (PKA) signaling pathway. (A) T cells were stimulated with 10 µg/ml LA for 10 min. Total protein was harvested and used for western blot analysis for Lck phosphorylation, which is a downstream effector of PKA signaling. The top blot is phospho-Lck and the bottom panel is Lck. (B) Graphical representation of densitometric analysis of band intensity showing the ratio of pLck/Lck. N = 9. *indicates statistical significance compared to untreated control (p<0.05).

We have shown that LA inhibits pro-inflammatory cytokine production and T cell activation. However, the biochemical mechanisms that mediate the anti-inflammatory actions of LA are not fully understood. We previously reported that LA stimulates cAMP production in inflammatory cells [9], [15]. cAMP is a second messenger that activates many signaling cascades, one of which is the Protein Kinase A (PKA) pathway [32]. PKA activation regulates a variety of eukaryotic cell functions, including T cell activation [33]. One established inhibitory PKA signaling pathway in T cells is PKA phosphorylation of Src kinase, which in turn phosphorylates and deactivates Lck, a protein required for T-cell receptor activation. To determine if LA induced cAMP production leads to stimulation of this inhibitory pathway, we examined the effects of LA on Lck phosphorylation. Western blot analysis revealed increased phospho-Lck (pLck) levels after treatment with LA for 10 minutes compared to untreated control (Fig. 4A, top panel). No changes were detected in total Lck levels in the absence or presence of LA. Densitometric analysis revealed that LA increased pLck levels by approximately 30% (Fig. 4B).

**Figure 5:**
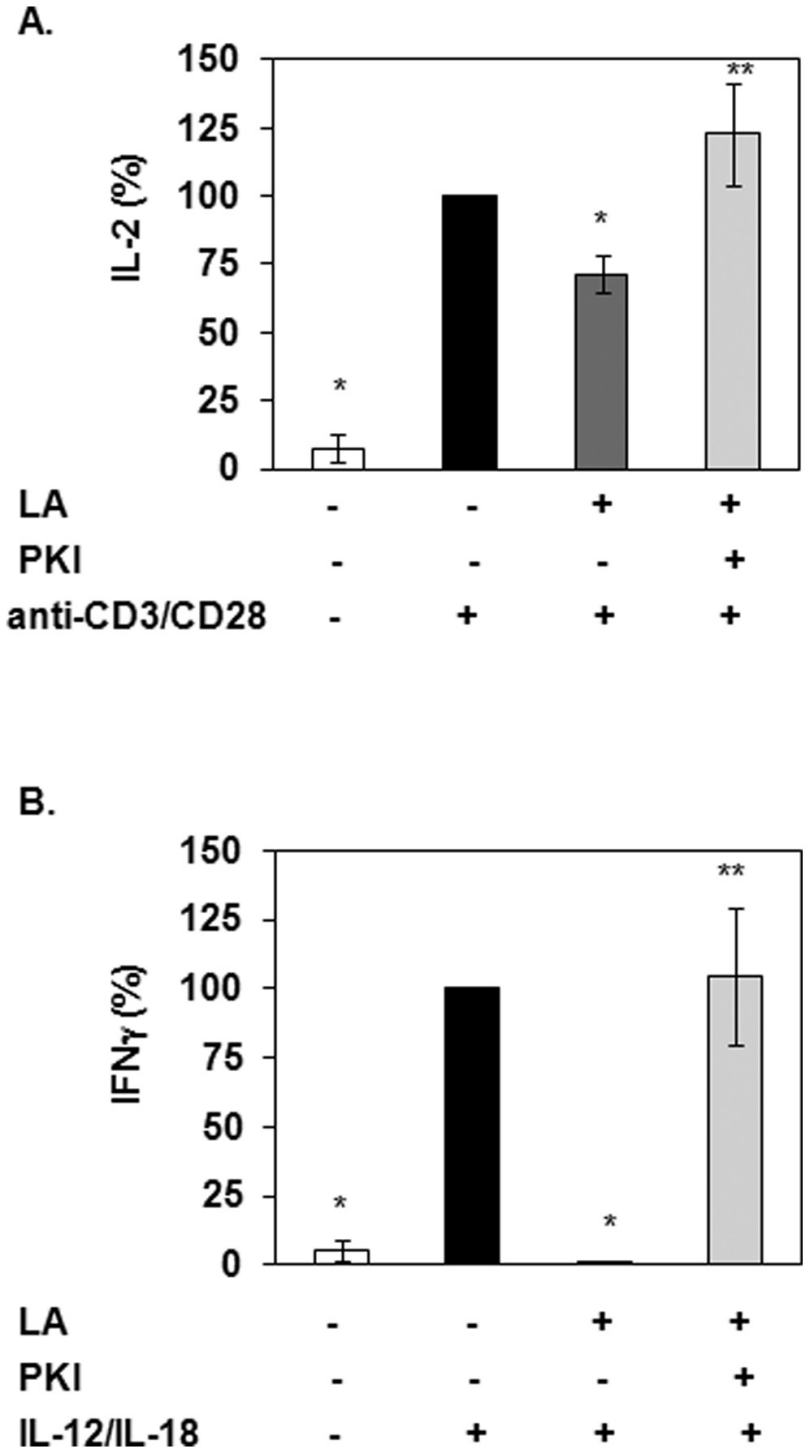
Pretreatment with PKI blocked the suppression of T cell and NK cell activation by LA. (A) T cell enriched PBMCs were incubated in 100 µM PKI for 30 min prior to treatment with 25 µg/ml LA for 5 min. Cells were then treated with 4 µg/ml anti-CD3/CD28 for 24 hours. Supernatants were collected and IL-2 levels were measured via ELISA (eBioscience, San Diego, CA). N = 3 donors in triplicate. *indicates statistical significance compared to anti-CD3/CD28 stimulated control. **indicates significance compared to LA plus anti-CD3/CD28 (*p*<0.05). (B) Purified NK cells were pretreated with 100 µM PKI for 30 min prior to treatment with LA for 5 min. Cells were then stimulated with 10 ng/ml IL-12/IL-18 for 6 hours. Supernatants were used to measure IFN gamma levels via ELISA (R&D systems, Minneapolis, MN). *indicates statistical significance compared to IL-12/IL-18 stimulated control. **indicates significance compared to LA plus IL-12/IL-18 (*p*<0.05).

We next determined if PKA mediates the effects of LA on T cell activation. T cell enriched PBMCs were incubated for 30 minutes with 100 µM PKI, a peptide that inhibits PKA by acting as a pseudosubstrate. Cells were then treated with 25 µg/ml LA for 5 minutes prior to stimulation with anti-CD3/CD28 for 24 hours. LA inhibited IL-2 production by 30% compared to stimulated controls and pretreatment with PKI blocked this inhibition (Fig. 5A). Treatment with PKI alone did not statistically alter anti-CD3/CD28 induced IL-2 production (data not shown).

To determine if the effects of PKI are specific to T cells, we conducted experiments in a different model looking at the effects of LA on NK cell activation. We previously reported that LA inhibits NK cell activation by blocking IFNγ production [9]. NK cells were incubated with PKI for 30 minutes prior to treatment with LA. Cells were then stimulated with 10 ng/ml IL-12/IL-18 for 24 hours and NK cell activation was determined by measuring IFNγ production. We showed that LA completely blocked IFNγ production compared to IL-12/IL-18 stimulated control (Fig. 5B). Similar to IL-2, pretreatment with PKI completely blocked IFNγ inhibition by LA. Collectively, the data demonstrates that LA activates the PKA signaling cascade to modulate T cell and NK cell activation.

### LA stimulates cAMP production in MS patients

**Figure 6:**
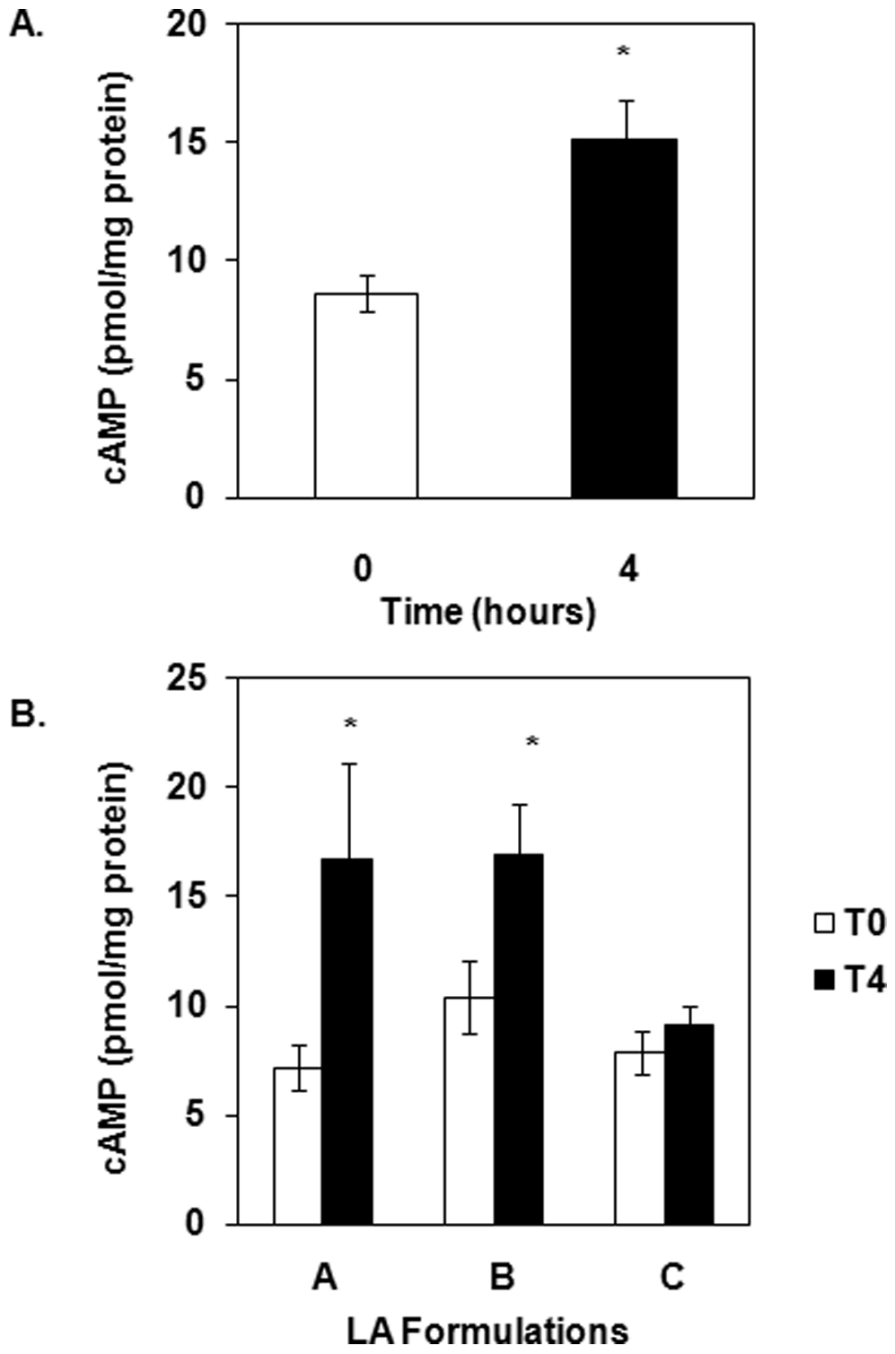
Oral administration of LA stimulates cAMP production in MS patients. (A) PBMCs were isolated from blood drawn from MS patients before (T0) and 4 hours (T4) after oral ingestion of 1200 mg racemic (50∶50 mixture of R and S enantiomers) LA. Previously frozen PBMCs were resuspended in 100 µl PBS. Fifty µl were transferred to new tubes then lysed in additional 350 µl 0.1 M HCl and boiled for 10 min. Samples were pelleted by centrifugation at 1300×g for 1 min. The supernatants were used for measuring cAMP levels via ELISA (Assay Designs, Ann Arbor, MI). N = 20 subjects in duplicate. *indicates statistical significance compared to T0 (*p*<0.05). (B) Subdividing (A) into groups according to the LA formulations received: formulation A, B or C. See Methods and Materials for a description of each formulation. *indicates statistical significance compared to T0 (*p*<0.05).

Up until this point, all experiments looking at the effects of LA on cAMP levels have been performed in cells *in vitro*. To determine if LA is able to stimulate cAMP production *in vivo* in human subjects with multiple sclerosis, thirty-six subjects were screened and 29 were randomized to receive 1 of 3 formulations of LA: A, B or C. The formulations differ in composition and were obtained from different companies (see materials and methods). Five subjects withdrew from the study, leaving 24 subjects who completed the study. See Table 1 for subject demographics (modified from [34]). Blood was drawn before (T0) and 4 hours after oral ingestion of LA (T4). A 1200 mg dosage of racemic LA was used (50∶50 mixture of R and S enantiomers). The 1200 mg dosage was found to be safe in a pilot study conducted by Yadav *et al.* [14]. Of the 24 remaining subjects, cAMP analyses on PBMCs were obtained from 20 subjects; the serum was contaminated for 1 subject and not enough cells were collected for the other 3 subjects. The average cAMP data for all 20 participants is shown in Fig. 6A. Baseline cAMP level (T0) was 8.65±0.79 pmol/mg protein (Table 1). Administration of LA resulted in a 43% increase in cAMP level (15.71 pmol/mg protein) compared to baseline. Figure 6B shows the cAMP data for each of the LA formulations administered. Formulation A, B and C resulted in increased cAMP levels by 57, 38.7 and 14.9%, respectively, compared to baseline. The data presented here is the first to show that oral administration of LA results in elevated cAMP levels *in vivo*.

**Table 1:** Baseline demographics and cAMP response in MS subjects.

		Formulation A	Formulation B	Formulation C	Total
**Number in group (n)**		8	8	8	24
**Gender**	Male	3	3	1	7
	Female	5	5	7	17
**Age (years)**	Median	48.5	52	50.5	51
	Range	36–66	27–77	42–65	27–77
**Race**	Caucasian	8	8	8	24
	Non-caucasian	0	0	0	0
**Type of MS**	Relapsing-remitting	4	3	5	12
	Primary progressive	1	0	0	1
	Secondary progressive	3	5	3	11
**MS duration (years)**	Median	11.5	21	11.5	12.5
	Range	1.0–16.0	8.0–33.0	2.0–35.0	1.0–35.0
**EDSS**	Median	4.0	5.0	4.0	4.0
	Range	2.0–7.0	2.5–7.5	2.0–8.0	2.0–8.0
**cAMP Response**	T0 (pmol/mg protein)	7.18±1.02 (6)	10.39±1.67 (7)	10.03±1.13 (7)	8.65±0.79 (20)
	T4 (pmol/mg protein)	16.72±4.4	16.93±2.36	10.04±0.63	15.18±1.71

Modified from [34]. For cAMP response, the numbers in parentheses indicate number of samples measured.

## Discussion

Inflammation is a critical component of MS involving T cells, antigen-presenting cells, cells of the innate immune system such as NK cells, and cells of the CNS such as microglia, astrocytes and neurons. These cells produce a cytokine milieu, including IL-6 and IL-17, which perpetuate the inflammatory response. IL-17 producing Th17 cells are now believed to be the major pathogenic T cell subset in EAE/MS and other autoimmune diseases [35], [36], although cause and effect relationships in human health have not been established. IL-6 is a pleiotropic cytokine with a variety of functions, but of importance for this study, IL-6 is an essential differentiation factor for Th17 cells [37], [38]. Both IL-6 and IL-17 mRNA have been shown to be highly expressed in MS lesions [39]. Elevated IL-17 levels in the serum and cerebrospinal fluid of MS patients have also been reported [40]. In EAE, wild-type mice developed encephalitogenic peptide pMOG 35–55 induced EAE, whereas IL-6 deficient mice are resistant to the disease [41]. Blockade of IL-6 using an anti-IL-6 receptor monoclonal antibody inhibited the development of EAE and the induction of myelin oligodendrocyte glycoprotein peptide-specific CD4-positive and Th17 T cells [42], indicating the protective effect of IL-6 blockade may be due to reducing the infiltration of autoreactive T cells into the CNS. In this study we show that LA inhibits IL-6 and IL-17 production in human PBMCs, suggesting that LA has the potential to be therapeutic in MS. It will be exciting to see if administration of LA to MS subjects will result in reduction in IL-6 and IL-17 levels in the serum and in circulating PBMCs.

To stimulate IL-6 production, we used LPS, which is a ligand for toll-like receptor 4 (TLR-4). The TLR-4 pathway leads to downstream activation of the transcription factor nuclear factor kappa B (NF-κB) and pro-inflammatory gene expression. How can LA, via cAMP/PKA signaling, affect the LPS/TLR-4 pathway? There are several reports supporting the idea that there is crosstalk between the two signaling pathways. Wall *et al.* demonstrated that cAMP/PKA inhibits NF-κB function by slowing down its translocation into the nucleus and preventing gene transcription [27]. Parry and Mackman also showed that cAMP/PKA inhibits NF-κB activity [43]. It is therefore possible that LA is inhibiting LPS induced NF-κB activity, thus reducing IL-6 production via activation of PKA. Although we show statistically significant decreases in IL-6 levels with LA treatment, it is important to note that a large amount of IL-6 is still produced in response to LPS stimulation (Fig. 1a) indicating that IL-6 can still modulate the inflammatory response in general and, specifically, the cellular response to toll-like receptor (TLR)-4 ligands. This may be advantageous since a common side effect of immunosuppressive drugs is increased susceptibility to infections. If IL-6 is available in the circulation, the host is still able to fight off infections.

LA has been shown to be therapeutic in EAE [10], [12], [44]. Several reports demonstrated inhibition of T cell migration *in vitro* and into the spinal cord of EAE animals treated with LA [10], [12]. However, very little is known about the effects of LA and its mechanisms of action in human diseases. LA has antioxidant properties where it scavenges reactive oxygen and nitrogen species and replenishes glutathione and other endogenous antioxidants [45]. More recently, we and others have shown that LA exhibits a non-redox anti-inflammatory role [7], [9], [10], [11], probably via modulation of various signaling cascades that mediate these processes. For instance, LA stimulates theproduction of the immunomodulator cAMP in human inflammatory cells via activation of the prostaglandin E2 (PGE_2_) EP2 and EP4 receptors [9], [15]. This is likely possible due to the fact that LA shares some structural similarities to PGE_2_ and they are both hydrophobic and can exist in multiple conformers. For detailed discussion, we refer readers to our previous publication [9].

In this study, we determined that LA decreased ^3^H-thymidine incorporation and IL-2 production, indicating that LA inhibits T cell proliferation and activation. This is consistent with other reports using an assortment of cAMP elevating agents [46], [47], [48], [49]. For example, pretreatment with cholera toxin, forskolin, PGE_2_ or dibutyryl-cAMP reduced IL-2 levels in murine CD4+ T cells [47]. Similarly, isoproterenol inhibited concanavalin A induced IL-2 secretion in T cells obtained from both asthmatic and non-asthmatic subjects [46]. Since MS/EAE is believed to be a T cell mediated disease, the ability of LA to inhibit T cell proliferation and activation may explain how LA can treat and prevent EAE and possibly MS.

In many autoimmune disease models, IL-10 has been shown to be protective [37], [50]. IL-10 is a homodimeric pleiotropic cytokine that is produced by numerous cell types including monocytes, macrophages, neutrophils and T cells [51] and can exert its anti-inflammatory effects on both myeloid and lymphoid cells [37]. IL-10 inhibits the development of Th1 type responses, exerts autocrine inhibitory effects on macrophages and DCs, and enhances the differentiation of IL-10 secreting T regulatory cells to provide a positive regulatory loop for its induction [51]. Here, we report that LA treatment has a biphasic effect on IL-10 secretion, although the data is not statistically significant. This is not a unique phenomenon. Franchimont *et al.* showed increased IL-10 levels using low concentrations of dexamethasone and decreased IL-10 levels to below baseline with higher doses of dexamethasone [52] The IL-10 promoter contains cAMP-responsive elements, which are bound by cAMP responsive element binding (CREB) protein, and myocyte enhancer factor (MEF)-2 binding sites. PHA stimulates T cells via cross-linking CD3 and the TCR complex [53], [54], which in turn activates MEF-2 [29]. cAMP leads to downstream activation of CREB. Binding of CREB or MEF-2 to the promoter results in increased IL-10 production [27], [29]. cAMP, however, has been shown to inhibit MEF-2 binding to the IL-10 promoter, thus inhibiting IL-10 synthesis [29]. It is possible that low LA concentrations stimulate CREB binding to the promoter to increase IL-10 production while high cAMP levels are interfering with MEF-2 binding, thus resulting in decreased IL-10 levels. Future studies will be undertaken to determine if this is true.

cAMP is a small molecule second messenger involved in activation of various signaling pathways. Generation of cAMP is mediated by activation of G-protein coupled receptors and subsequent activation of transmembrane and soluble adenylyl cyclases (tmAC and sAC, respectively) [55], [56], [57]. cAMP, in turn, activates downstream signaling cascades, the most studied being the serine/threonine protein kinase, PKA, pathway. PKA is ubiquitously expressed and is composed of four subunits: two catalytic subunits and a dimer of regulatory subunits [33]. Upon binding to cAMP, the regulatory subunits are released from the catalytic subunits, and thereby activated. In T cells, PKA can inhibit cell activation by phosphorylating C-terminal Src kinase (Csk) which phosphorylates and deactivates Lck, a protein necessary for T cell receptor activation [32], [33]. Our data suggest that LA activates PKA leading to Lck phosphorylation and deactivation. Employing a different method, Marracci *et al.* showed that LA treatment dissociates Lck from CD4 in Jurkat cells leading to down-modulation of surface CD4 expression [7].

**Figure 7:**
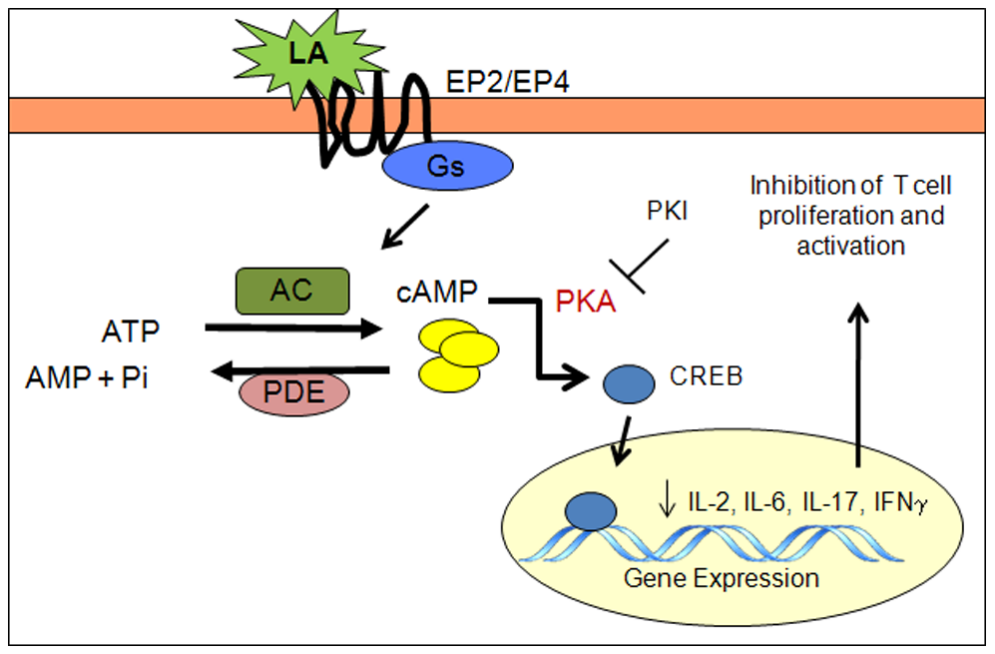
Schematic illustration of the putative LA signaling events. LA binds to the EP2 and EP4 receptors to activate the Gs subunit of the trimeric G-proteins. Gs activates adenylyl cyclase (AC), which generates cAMP from ATP. AC is degraded by phosphodiesterases (PDEs) into AMP and Pi. cAMP activates the PKA signaling cascade leading to inhibition of immune cell function. PKI acts as a pseudosubstrate to inhibit PKA signaling.

To confirm that PKA mediates the effects of LA, experiments were performed to determine the effects of blocking PKA activation on LA function. A variety of inhibitors are available that can be used to inhibit PKA activity. In this study we used PKI, a 21 amino acid peptide which acts as a pseudosubstrate of PKA. Pretreatment of T cells with a cell permeable PKI peptide prior to incubation with LA and stimulation with anti-CD3/CD28 blocked the attenuation of IL-2 production (T cell activation) by LA. Similarly, PKI ameliorated LA mediated suppression of IFNγ synthesis in NK cells indicating that LA induced activation of PKA is not confined to T cells. These data show that LA modulation of immune cell function is mediated in part via activation of PKA signaling. In figure 7, we illustrate the molecules that we have shown to mediate the effects of LA. LA binds the EP2/EP4 receptors to activate tmACs, which in turn generate cAMP. cAMP leads to activation of PKA signaling and inhibition of immune cell function.

The *in vitro* data demonstrating modulation of cAMP production and the immune response by LA is now supported by *in vivo* data. This study is the first to show the effects of LA on cAMP production *in vivo*. We demonstrated that a 1200 mg dose of LA given orally increased average cAMP levels by 43% in PBMCs obtained from subjects with MS. This dosage is well tolerated and considered safe [14]. Subdividing the data into groups according to the different formulations given illustrates that formulation A and B elicited a robust increase in cAMP (57 and 38.7% increase, respectively), whereas the increase in cAMP levels resulting from ingestion of formulation C were more modest (14.9% increase). It is possible that the difference in response is due to varying absorption rates of LA. A recent publication analyzing the pharmacokinetics of serum LA concentration in the same subjects presented in this study demonstrates that serum LA concentrations peaked at an earlier time point (about 30 minutes faster) for formulation C compared to formulations A or B [34]. Since absorption rate is determined by the rate of dissolution of a compound, its formulation may influence this process depending on the conditions of the gastrointestinal environment, such as pH, intestinal flora and enzyme content [58]. Furthermore, because cAMP levels are transient, four hours post ingestion may have been too late to detect increased cAMP levels for formulation C. Future studies looking at the kinetics of cAMP levels at the same time points used for determining the pharmacokinetics of LA will allow us to better correlate the peak cAMP response to peak serum LA concentration.

Components other than LA are present in the tablets/capsules being ingested. Is it possible that these additives are regulating cAMP production? While we cannot eliminate other components, it is unlikely that ascorbic acid, which is the most abundant additive in formulations B and C, is responsible for the elevation in cAMP levels. *In vitro* studies in PBMCs indicate that ascorbic acid treatment does not result in increased cAMP production (unpublished data). Future studies utilizing the appropriate placebo controls will determine that cAMP production is a specific response to LA treatment.

Administration of LA is therapeutically effective in diabetic polyneuropathy and other diseases [59], [60], [61]. A pilot study in MS patients demonstrated that oral administration of LA for two weeks resulted in decreased amounts of matrix metalloproteinase-9 and soluble ICAM-1, markers of inflammatory activity, in the serum [14]. The ability of LA to stimulate cAMP production is also encouraging since cAMP and components of its signaling cascade have been therapeutic targets. For instance, phosphodiesterase inhibitors such as milrinone, which prevents the breakdown of cAMP thereby increasing its intracellular concentration, is used to treat coronary heart disease [62]. Forskolin, a non-specific cAMP elevating agent produced from the plant *Coleus forskohlii*, is also being pursued for medicinal purposes. Cell culture and animal studies have shown that forskolin has anti-inflammatory effects similar to LA. For instance, forskolin and its derivatives inhibit T cell proliferation and activation, as well as attenuate proinflammatory cytokine production [63], [64], [65]. However, it has not yet been determined if forskolin is able to stimulate cAMP production *in vivo* or if forskolin is bioloigically available since it has low water solubility. LA, on the other hand, is biologically available *in vivo* [*45*]. LA is also considered safe, is produced endogenously in human beings and has antioxidant properties that may contribute to its beneficial therapeutic effects [14], [45].

In summary, we show that LA exhibits anti-inflammatory properties by inhibiting IL-6 and IL-17 production and T cell proliferation and activation while possibly maintaining IL-10 synthesis. In addition, LA activates the PKA signaling pathway. We also show for the first time that oral ingestion of LA tablets/capsules lead to elevated cAMP levels *in vivo*. These data provide a foundation for future studies, both *in vitro* and in MS patients, to determine the therapeutic potential of LA.

## Materials and Methods

### Materials and Reagents

RPMI, fetal bovine serum and all other tissue culture reagents were purchased from Invitrogen (Carlsbad, CA). The EasySep NK cell negative purification kit was purchased from Stem Cell Technologies Inc. (Vancouver, British Columbia). The cAMP kits were purchased from BioAssay Designs (Ann Arbor, MI). The BCA protein assay kit was obtained from Pierce Biotechnology (Rockford, IL). ELISA kits/reagents were purchased from R&D Systems (Minneapolis, MN) or Ebioscience (San Diego, CA). LA for in vitro studies was purchased from Sigma (St. Louis, MO). For *in vivo* studies, LA was obtained from three sources: Viatris (formulation A), Vital Nutrients (formulation B) or Pure Encapsulations (formulation C). Pure Encapsulations® (Sudbury, MA, USA) provided vegetable capsules containing 600 mg LA (Lot # 3480504 expiration 11/06). Each 600 mg LA capsule contained 30 mg of vitamin C (ascorbyl palmitate derived from corn dextrose fermentation) and pine cellulose plant fiber to add to volume. Vital Nutrients® (Middletown, CT, USA) provided gelatin capsules containing 300 mg LA (lot # 5G24 expiration 08/07). Each also contained cellulose powder (may contain Ascorbyl Palmitate and/or Silica). Viatris® (now called Meda Pharma®) LA was a tablet formulation containing 600 mg LA (lot # 4E002-1, expiration date 05/07).

### Human Subjects

The study received approval from the Oregon Health & Science University (OHSU) Institutional Review Board prior to initiation. All subjects gave written informed consent prior to participation in the study. To qualify, subjects needed to meet the following inclusion/exclusion criteria: definite MS by McDonald's [66] or Poser's criteria [67], EDSS≤7.5 [68]; age 18–70 inclusive; no clinically significant MS exacerbation within 30 days of the screening; no systemically administered corticosteroids within 30 days of study entry; female subjects were not pregnant or breast-feeding; no LA in previous 2 weeks; subjects were not on anti coagulants such as heparin or coumadin or aspirin during the study; and subjects did not have other significant health problems (e.g. active coronary heart disease, liver disease, pulmonary disease, diabetes mellitus) that could increase risk of experiencing adverse events or was unable to give informed consent. Subjects were allowed to receive symptomatic medications while on study. Subjects who took aspirin were asked to stop aspirin 7 days prior to the study. Subjects taking recombinant interferon (IFN)-b or glatiramer acetate were allowed to continue taking these medications. Subjects were randomized to receive one of three treatment arms: formulation A, B or C. Blood draws were obtained for subjects at baseline (T = 0) and four hours after oral ingestion of LA (T = 4). Cells and serum were separated via centrifugation. PBMCs were isolated from total cells through ficoll purification similar to steps described below. PBMCs were aliquoted and frozen down in liquid nitrogen for future use.

### Cell culture

Human PBMCs were obtained from source leukocytes (buffy coat) from the Red Cross in Portland, OR (approval #VACARR) or apheresis products purchased from Keybiologics (Memphis, TN). For buffy coats, enriched leukocytes were subjected to ficoll purification (Amersham) and centrifugation at 1400 rpm with the brake turned off for 30 minutes to remove contaminating red blood cells and platelets. The interface was collected, washed with RPMI 1640 and centrifuged at 1300 rpm for 10 minutes. The supernatant was decanted and the cells were subjected to two more wash steps. Cells were resuspended in freezing medium (RPMI+25% FCS+12% DMSO) and stored in liquid nitrogen for future use. For apheresis products, blood was split into conical tubes, diluted with 4× volume with 1× PBS (no Ca^2+^ or Mg^2+^), and centrifuged at 200×g for 15 min at RT. Supernatants were pipetted off the top (pellet is very loose), cells were resuspended in a small volume of PBS by flicking the tube, and fresh PBS (50 ml) was added. Cells were centrifuged at 200×g for 15 min at RT. This wash step was repeated once more. Cells were then subjected to ficoll gradient purification and frozen down as described above for buffy coats.

Previously frozen PBMCs were thawed and NK cells purified using the EasySep negative purification kit following the manufacture's protocol. Briefly, human NK cell enrichment cocktail (50 µl/ml cells) was added to PBMCs (2×10^7^ cells/ml) in PBS+2% FBS and 1 mM EDTA and incubated at RT for 10 min. EasySep magnetic microparticles (100 µl/ml cells) were added to the cell mixture and incubated at RT for 5 min. The total suspension was brought up to 2.5 mls with buffer and placed into a magnet for 2.5 minutes. The supernatant containing the purified NK cells was then collected for experiments.

### ELISA

T cell enriched PBMCs (2×10^6^) were pretreated with the indicated concentrations of LA for 5 minutes and then stimulated in 14 ml falcon polypropylene tubes to determine IL-6, IL-10, IL-17 and IL-2 levels via ELISA. For IL-6, cells were treated with 5 µg/ml LPS for 6 hours. A combination of 4 µg/ml anti-CD3 and 2 µg/ml anti-CD28 was used to stimulate cells for 24 hours for IL-17. Two µg/ml PHA was used to stimulate IL-10 production for 24 hours. For IL-2, cells were stimulated in tubes coated with anti-CD3 (4 µg/ml OKT3) and soluble anti-CD28 (5 µg/ml BD Pharmingen, Los Angeles, CA). Prior to stimulation, cells were exposed to LA for one minute, rinsed and then added to the tubes for stimulation. Supernatants were collected for each treatment and used to measure cytokine levels using ELISA kits purchased from R&D Systems (Minneapolis, MN) or Ebioscience (San Diego, CA) following the manufacturer's protocol. Endpoint reading was determined by measuring the absorbance at 405 nm on a colorimetric plate reader using Softmax Pro software (Molecular Devices, Sunnyvale, CA).

### Proliferation Assay

Proliferation assay was measured by [^3^H]-thymidine incorporation. T cell enriched PBMCs (7.5×10^4^) were pretreated with 10 or 50 µg/ml LA for one minute prior to stimulation with immobilized anti-CD3 and soluble anti-CD28. We previously showed that we can achieve maximal LA stimulation of cAMP production as early as 1 minute, with cAMP levels decreasing after 5 minutes [15]. After 48 hours of stimulation 10 µl [^3^H]-thymidine (50 µCi/ml) was added to the cells. 16 hours later cells were collected and washed using a Tomtec cell harvester and glass fiber membrane (Wallac). Incorporated tritium was determined by scintillation counting.

### Lck Phosphorylation

Two million PBMC were incubated with LA for one minute, centrifuged (1350×g 60s), rinsed with 500 µl RPMI 1640, and centrifuged (13,000×g 60s). Cells were then lysed by boiling with 30 µl boiling SDS gel loading sample buffer. Ten µl of total cell extract was subjected to SDS-PAGE and transferred to PVDF membrane (Immobilon-P, Millipore). Membranes were blocked with 5% milk TTBS for Lck Westerns and 3% BSA TTBS for Y-505-pLck antibodies (Transduction laboratories, BD Pharmingen, San Diego, Ca). HRP-conjugated goat anti-mouse (Santa Cruz Biotech, Santa Cruz, CA) and Perkin-Elmer chemiluminescence were used for detection (Boston, MA).

### PKI Inhibition Assay

Poly-R-PKI (Biomatik Corporation, Wilmington, DE) was used to determine if PKA signaling mediates the effects of LA on NK cell and T cell activation. Poly-R PKI is a cell permeable peptide inhibitor with the sequence GRTGRRNAIRRRRRRRRRRRR. It acts as a pseudo-substrate that binds to the catalytic subunits of PKA. Stocks were made by dissolving powder into water. For NK cell activation, we measured IFNγ production. NK cells (1×10^5^) were pretreated with 100 µM PKI for 30 minutes then treated with 50 µg/ml LA for 5 minutes. Cells were stimulated with a mixture containing 10 ng/ml IL-12 and IL-18. Ten percent serum was added 30 minutes after stimulation and incubated at 37°C for a total of 24 hours. IFNγ levels were determined by ELISA (R&D Systems, Minneapolis, MN). A 96-well plate was coated with 100 µl αIFNγ capture antibody (MAB2852) at 4 µg/ml and incubated for 2 hours at 37°C. Wells were washed 5 times with TTBS, blocked with 3% BSA/TTBS (200 µl) for 1 hour at RT and washed again. Recombinant human IFNγ standards (285-IF100) or treated sample supernatants were added to each well (100 µl) and incubated at RT for 2 hours. Wells were washed with TTBS 5× and incubated with 100 µl biotinylated αIFNγ (200 ng/ml) (BAF285) for 2 hours at RT. After washing, wells were incubated with 100 µl streptavidin-HRP for 20 minutes at RT in the dark. Wells were washed with TTBS and incubated with 100 µl TMB substrate (52-00-01) for 20 minutes at RT in the dark. The reaction was stopped with the addition of 50 µl 1M H_2_SO_4_ and absorbance was measured at 450 nm.

For IL-2, T cell enriched PBMCs (2×10^6^) were pretreated with 100 µM PKI for 30 minutes at 37°C. Cells were treated with 25 µg/ml LA for 5 minutes then stimulated with 4 µg/ml soluble, human anti-CD3 (Zymed, San Francisco, CA) and anti-CD28 (Ancell, Bayport, MN) for 24 hours at 37°C. Ten percent serum was added 30 minutes after stimulation. Supernatants were collected after brief centrifugation and used for IL-2 ELISA following manufacturer's protocol (Ebioscience, San Diego, CA). Absorbance reading was measured at 405 nm.

### Cyclic AMP assay

Previously frozen PBMC obtained from human subjects (between 5–7 million cells) were used for analysis of cAMP levels. Pellets were resuspended in 100 µl cold 1× PBS on ice. Half of that volume (50 µl) was transferred to a new tube containing 350 µl 0.1 M HCl. Cells were lysed by boiling for 10 minutes. Samples were centrifuged at 1300 rpm and 100 µl of supernatants were used for cAMP assays following the manufacture's protocol. The absorbance was measured at 405 nm using a colorimetric 96-well plate reader. Results in pmol/ml were then divided by the protein concentration (see BCA assay below) to obtain pmol of cAMP per milligram of protein.

### Bicinchoninic acid (BCA) assay

Ten microliters of supernatants from cAMP assays were used to determine total protein concentrations using the BCA assay kit (Pierce, Rockford, IL) following the manufacturer's protocol. Absorbance readings were measured at 562 nm. Bovine serum albumin (BSA) standards were prepared in 0.1M HCl at concentrations ranging from 0–1 mg/ml. Protein concentrations for unknown samples were extrapolated from the standard curve using Softmax Pro software (Molecular Devices, Sunnyvale, CA).

### Statistical analysis

The data were analyzed using EXCEL 2007. The mean and standard error of the mean were calculated. Statistics were performed using Student's *t*-test and were considered significant at a *p* value of ≤0.05. All treatments were performed independently at least 3 times.
